# The change of health-related quality of life after minimally invasive esophagectomy for esophageal cancer: a meta-analysis

**DOI:** 10.1186/s12957-018-1330-9

**Published:** 2018-05-24

**Authors:** Yong Zhang, Xiaomei Yang, Donghong Geng, Yingfei Duan, Junke Fu

**Affiliations:** 1 Department of Thoracic Surgery, the First Affiliated Hospital of Xi’an Jiaotong University, No. 277 West Yanta Road, Xi’an, 710061 China; 2 Hospital 521 of China’s Ordnance Industry Group, Xi’an, 710065 China; 3 School of Continuing Education of Xi’an Jiaotong University, Xi’an, 710061 China; 4 Department of Pathology, the First Affiliated Hospital of Xi’an Jiaotong University, Xi’an, 710061 China

**Keywords:** Esophageal cancer, Health-related quality of life (HRQL), Minimally invasive esophagectomy (MIE), Meta-analysis

## Abstract

**Background:**

Short- and long-term health-related quality of life (HRQL) was severely affected after surgery. This study aimed to assess the direction and duration of HRQL from 3- to 24-month follow-ups after minimally invasive esophagectomy (MIE) for esophageal cancer.

**Methods:**

A systematic literature search in MEDLINE, EMBASE, and the Cochrane database was performed for all potentially relevant studies published until February 2017. Studies were included if they addressed the question of HRQL with OERTC-QLQ-C30 and OES18. Primary outcomes were HRQL change at 3-month follow-up. Secondary outcomes were HRQL change from 3-, 6- (short-term) to 12- (mid-term), and/or 24-month (long-term) follow-ups.

**Results:**

Six articles were included to estimate the change in 24 HRQL outcomes after MIE. Most of the patients’ HRQL outcomes deteriorated at short-term follow-up and some lasted to mid- or long-term after MIE. Patients’ physical function and global QOL deteriorated from short- to long-term follow-ups, and emotional function had no change. The directions of dyspnea, pain, fatigue, insomnia, constipation, diarrhea, cough, and speech problems were increased. The deterioration in global function lasted 6 months, the increase in constipation and speech problems lasted 12 months, and insomnia increased more than 12 months after MIE.

**Conclusions:**

The emotional function had no change after MIE. The global QOL become worse during early postoperative period; the symptoms of constipation, speech problems, and insomnia increased for a long time after MIE.

## Background

Esophageal cancer is one of the most common malignant tumors of the digestive system with a poor prognosis with overall 5-year survival rates only 15–50%, and the incidence of esophageal cancer has risen steadily during recent decades [1–3]. Esophagectomy with lymphadenectomy is regarded as the only curative option for patients with resectable esophageal cancer [4–6].

The traditional open esophagectomy (OE) is a relatively high invasive surgery, which may lead to several morbidities or prominent mortality [7]. Minimally invasive surgery is assumed to reduce surgical injury and improve patients’ prognosis. With the developing skills and increasing experiences in laparoscopy and thoracoscopy in thoracic and stomach surgery, minimally invasive esophagectomy (MIE) has become the recommended approach, popularized in centers with experienced surgeons [8].

A lot of longitudinal and meta-analysis studies have been performed to compare the outcomes of OE with MIE, which conclude that MIE is a safe alternation or better choice for esophageal cancer to OE because patients undergoing MIE may benefit from shorter hospital stay, lower complications, less morbidity, and overall survival [9–12]. Studies that investigate health-related quality of life (HRQL) after surgery for esophageal cancer show that patients will experience an impaired quality of life post operation, and MIE had an overall benefit on quality of life (QOL) for the patients compared with open surgery [13–20]. However, few studies focus on assessing the impact of MIE for esophageal cancer on HRQL and the change of HRQL after surgery [13, 18]. On this basis, the aim of this meta-analysis is to analyze the change of short- to long-term QOL after MIE for esophageal cancer.

## Methods

### Search strategies

A MEDLINE, EMBASE, and Cochrane database search was performed by two authors on all relevant clinical studies published until February 2017, analyzing quality of life after minimally invasive esophagectomy for esophageal cancer. The following keywords and medical subject headings were used: esophageal neoplasms, esophageal cancer, esophagus cancer, esophagus carcinoma, oesophageal cancer, esophageal carcinoma, oesophageal carcinoma, cancer of esophagus, carcinoma of esophagus, esophagectomy, resection of esophagus, minimally invasive surgical procedures, minimally invasive surgery, video-assisted thoracic surgery, thoracoscopic, thoracoscopy, laparoscopic, laparoscopy, quality of life, life quality, living quality, and quality of lives. Only studies on humans and written in English were considered for inclusion. The related-articles function was used to expand the search from each identified relevant study. A manual cross-reference search from articles was also performed. All citations and abstracts identified were thoroughly reviewed. The latest date for this search was 20 February 2017. Data quoted as unpublished or data from abstracts were not used. Any disagreements regarding which studies should be included that existed in two researchers were resolved through discussion.

### Inclusion and exclusion criteria

Studies were selected if they reported on a series of patients who underwent minimally invasive esophagectomy because of esophageal cancer. Procedures of minimally invasive esophagectomy included thoracoscopy combined with laparotomy or laparoscopy.

All studies included in this meta-analysis also required to present detailed information used to assess quality of life and on when the questionnaire was administered. Only those were selected when all patients filled out the questionnaires before operation and at the follow-up (3, 6, 12, and/or 24 months after operation) by letter visit or out-patient consultant. Questionnaires that were used to analyze HRQL included, but not limited to, European Organization for Research and Treatment of Cancer (EORTC) QLQ C30 and OES18. Those that only presented their results graphically were excluded. When studies were discovered to report (partially) similar patient data, only the most recent and complete data sets were included.

### Data extraction

Data was extracted independently by two reviewers (XM Yang and YF Duan) from each study: study characteristics (first author, year of publication, study design, study aim, timing of follow-up and HRQL data gathering, and type of questionnaire used), population characteristics (number of patients included, demographics, cancer histologic type, cancer stage, cancer site, and neoadjuvant therapy), item, and total results.

We contacted the first or corresponding author of each article by e-mail if not all descriptive outcome data was reported. If necessary, we used reported 95% confidence intervals (95% CIs), standard errors, to transform missing SD data [21].

### Interested outcomes

The primary outcome of this study was HRQL change at 3-month follow-up. The second outcome was HRQL change from 3-, 6- (short-term) to 12- (mid-term), and/or 24-month (long-term) follow-ups.

The studies were included when both the following validated quality of life instruments were used: the EORTC-QLQ-C30 and the EORTC-QLQ-OES18. The QLQ-C30 questionnaire was developed by the Quality of Life Division of EORTC. It explored the generic quality of life of patients affected by oncologic diseases and includes a global health status scale, five functional scales (physical, role, emotional, cognitive, and social), three symptom scales (fatigue, nausea and vomiting, and pain), and six single items (dyspnea, insomnia, anorexia, constipation, diarrhea, and financial difficulties) [13]. The QLQ OES-18 assessed symptoms specific to esophageal cancer and was composed of four symptom scales (dysphagia, eating, reflux, and esophageal pain) and six single items (swallowing saliva, choking when swallowing, dry mouth, taste problem, coughing, and speech problem) [14]. Each item had four response alternatives: “not at all” (scored as 1), “a little” (scored as 2), “quite a bit” (scored as 3), and “very much” (scored as 4), except for the global QOL scale which ranges from “very poor” (scored as 1) to “excellent” (scored as 7). All QLQ-C30 and QLQ-OES18 responses were transformed linearly to scores ranging from 0 to 100.

### Statistical analysis

Review Manager Version *5.3* (Copenhagen: the Nordic Cochrane Centre, The Cochrane Collaboration, 2014) was used to perform meta-analysis. The data can be synthesized only when the number of studies exceeds two. Measurement data reported as mean SD/SE were adopted. The results were presented as weighted mean differences [95% confidence interval (CI)]. Heterogeneity was assessed by *χ*^2^ and *I*^2^. An *I*^2^<30% represented low heterogeneity, 30–50% moderate, 50–75% substantial, and 75–100% considerable heterogeneity [7, 22]. The statistical results used fixed-effect models for low and moderate heterogeneity, random-effect models for substantial and considerable heterogeneity. The study design and risk of bias are shown in Table 1. The definitions of direction, clinical relevance, and duration of HRQL change were same as those described by Jacobs et al. [13].

**Table 1:** The study design and risk of bias of studies included in the meta-analysis

The first author	Study design	Risk of bias						
		Sequence generation	Allocation concealment	Blinding patient/personnel	Incomplete outcome data	Blinding outcome	Selective outcome reporting	Other source of bias
Barbour et al. [17]	Cohort	High	High	High	Unclear	High	Low	Low
Wang et al. [19]	Cohort	High	High	High	Unclear	High	Low	Low
Maas et al. [20]	RCT	Low	High	High	Low	High	Low	Low
Wang et al. [15]	Cohort	High	High	High	Unclear	High	Low	Low
Parameswaran et al. [16]	Case series	N/A	N/A	N/A	N/A	High	Low	Low
Nafteux et al. [18]	Cohort	High	High	High	Unclear	High	Low	High

## Results

### Selected studies

**Fig. 1:**
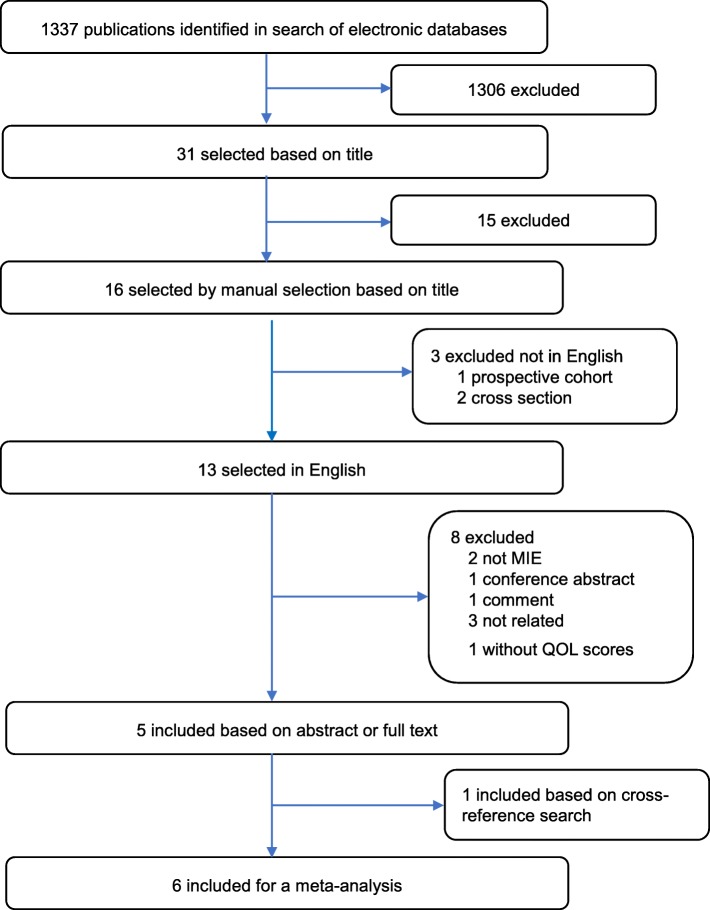
Flowchart of literature identification and screening process

A flowchart of the literature screening process is shown in Fig. 1. The initial search yielded 1337 articles, of which 1306 were excluded based on their titles. Fifteen duplicated articles were then manually excluded on the basis of their titles. Three articles were excluded because of not being written in English. Eight articles were further excluded on the basis of their abstracts or full texts, of which two were conference abstracts [23, 24]; one was comment [25], one was systematic review [14], two presented their results only in graphical formats [26, 27], one presented their results only in percent of patients [28], one was related to the reliability and validity of the questionnaire [22], and one article was included based on the cross-reference search [17]. Six articles finally met the criteria of inclusion [15–20], and two reported data form the same hospital by the same first author [15, 19]. Although they reported the same dataset, two studies from Zhongshan Hospital were included in the analysis because one analyzed more aspects of HRQL [15, 19]. However, all the patients included in these studies were counted only once, and only the most recent and complete data sets were selected.

### Characteristics of studies and patients

The selected trials included a total of six studies that were published between 2010 and 2016 (Table 2), of which two were retrospective studies [15, 19], three were prospective ones [16–18], and only one was a randomized controlled trial (RCT) [20]. One study was done in Australia [17], one in Belgium [18], two in China [15, 19], one in the UK [16], and the last one in the Netherlands [20]. Five studies used both EORTC QLQ C30 and the disease-specific OES18 questionnaires. One study was performed not only using EORTC QLQ C30 and the disease-specific OES18, but also SF36. The HRQL was the primary outcome for all studies. Patients were all enrolled consecutively. The measure times of HRQL were preoperation (baseline), 3-, 6-, 12-, and/or 24-month postoperation. One study aimed to assess HRQL after MIE [16], and five studies aimed to compare HRQL in patients after MIE and open esophagectomy [15, 17–20].

**Table 2:** Studies characteristics: study setting and feature

Study	Year	Country	Center	Randomized	Prospective data	Consecutive	HRQL as primary outcome	Preoperative HRQL assessment	SF36	OES18	QLQ C30	Timing HRQL measures
Barbour et al. [17]	2016	Australia	Princess Alexandra Hospital	No	Yes	Yes	Yes	Yes	No	Yes	Yes	Baseline, 3,6,9,12,18,24 mo po
Wang et al. [19]	2015	China	Zhongshan Hospital	No	Yes	Yes	Yes	Yes	No	Yes	Yes	Baseline, 1, 3,6,12,18,24 mo po
Maas et al. [20]	2015	Netherlands	VU University Medical Center	Yes	Yes	Yes	Yes	Yes	Yes	Yes	Yes	Baseline, 1.5, 12 mon po
Wang et al. [15]	2010	China	Zhongshan Hospital	No	No	Yes	Yes	Yes	No	Yes	Yes	Baseline, 0.5, 1, 4 6 mon po
Parameswaran et al. [16]	2010	UK	Royal Devon and Exeter NHS Foundation Trust	No	Yes	Yes	Yes	Yes	No	Yes	Yes	Baseline, 1.5, 3, 6, 12 mon po
Nafteux et al. [18]	2011	Belgium	UZ Gasthuisberg	No	Yes	Yes	Yes	Yes	No	Yes	Yes	Baseline, 1–3, 4–6, 10–12 mon po

The number of patients included in the selected trials was 1034, of which 848 were males. The range of mean ages reported by different papers was 56 to 67 years. The indication for surgery was esophageal adenocarcinoma in 442 patients, squamous cell carcinoma in 576 patients, and others in 16 patients. The pathological TNM stages were stage 0 and stage I in 279 cases, stage II in 444 cases, stage III in 262 cases, and stage IV in 36 cases. All patients received neoadjuvant chemo- or chemoradiotherapy in one study [20]; no patients received in two studies [15, 18]; and partial patients received in three studies [16, 17, 19]. All patients received a combination of general and epidural anesthesia during the operation. In the first 3–5 days after surgery, patients received epidural or intravenous analgesia. The follow-up duration after esophagectomy, as reported in the articles, was between 6 and 72 months. The characteristics of the patients included in each study are described in Table 3.

**Table 3:** Patient characteristics

Study	Recruitment period	Intervention	Cases	M/F	Median/mean age (range)	Pathology (adeno/SCC/other)	TNM stage	Tumor site (U/M/L/GEJ)	ASA grade	Mean/median follow up (months)	Neoadjuvant treatment (no/CT/CRT)
Barbour et al. [17]	1988–2011	TAMK	377	316/61	64 (27–84)	299/78/0	51/80/125/121/0(0/I/II/III/IV)	0/42/299/36	275/102 (1&2/3&4)	27	175/87/115
Wang et al. [19]	2004–2013	MIE ^†^	444	362/82	56 (32–77)	0/444/0	62/254/100/28 (0&I/II/III/IV)	63/244/137/0	321/107/16 (1/2/3)	27	364/22/58
Maas et al. [20]	2009–2011	MIE ^†^	59	43/16	62 (34–75)	35/24/0	1/4/26/11/4/9/4 (0/I/II/III/IV/No residual tumor of LN. matas./no surgery)	N/A	10/34/14/1 (1/2/3/4)	12	0/5/54
Wang et al. [15]	2007–2008	MIE ^‡^	27	19/8	60.7 ± 9.3	1/25/1	6/14/4/3/0 (0&I/IIa/IIb/III/IV)	4/17/6//0	N/A	6	27/0/0
Parameswaran et al. [16]	2005–2007	MIE ^‡^	62	56/6	67 (49–80)	55/5/2	3/7/21/27/4 (0/I/II/III/IV)	N/A	N/A	12	0/48/0
Nafteux et al. [18]	2005–2010	MIE ^‡^	65	52/13	63.1(41–82)	52/N/A/13	10/55/0/0/0 (0/I/II/III/IV)	N/A	N/A	72	65/0/0

*TAMK* thoracoscopically assisted McKeown esophagectomy, *MIE* minimally invasive esophagectomy, *Adeo* adenocarcinoma, *SCC* squamous cell carcinoma *N/A* not applicable

^†^Thoracosopy + laparoscopy or laparotomy + cervical or thoracic anastomosis

^‡^Thoracosopy + laparoscopy or laparotomy + cervical anastomosis

### HRQL change

Twenty-four HRQL outcomes were included in our meta-analysis. Mean difference with 95% confidence intervals are reported in Table 4, which also reflects the average HRQL change.

**Table 4:** Change in 24 HRQL outcomes after MIE at 3,6,12 and 24 months follow-up

	Baseline vs. 3 months					Baseline vs. 6 months					Baseline vs. 12 months					Baseline vs. 24 months				
	Patients^†^	MD	Heterogeneity			Patients^†^	MD	Heterogeneity			Patients^†^	MD	Heterogeneity			Patients^†^	MD	Heterogeneity		
	(groups)	(clinical relevance)	Tau^2^	Chi ^2^	I^2^(%)	(groups)	(clinical relevance)	Tau^2^	Chi ^2^	I^2^(%)	(groups)	(clinical relevance)	Tau^2^	Chi ^2^	I^2^(%)	(groups)	(clinical relevance)	Tau^2^	Chi ^2^	I^2^(%)
Physical function ^‡^	1753	-14.36 [-11.77, -16.95]	2.88	P=0.09	58	1841	-9.32 [-5.36, -13.28]	11.17	P=0.001	81	1787	-7.42 [-4.80, -10.03]	3.29	P=0.10	53	1489	**-4.31 [-3.27, -5.36]**	**1.87**	**P=0.17**	**46**
	3	Medium				4	Small				4	Small				2	**Trivial**			
Role function ^‡^	919	-18.55 [-3.14, -33.97]	175.01	P<0.00001	95	1007	-10.01 [3.93, -23.94]	187.29	P<0.00001	95	899	-9.07 [0.04, -18.18]	48.02	P=0.01	77	N/A	N/A	N/A	N/A	N/A
	3	Medium				4	Small				3	Small								
Emotional function ^‡^	919	0.83 [8.45, -6.79]	36.33	P=0.003	82	1007	3.47 [8.88, -1.94]	19.65	P=0.02	68	**899**	**2.05 [6.05, -1.95]**	**1.78**	**P=0.32**	**11**	N/A	N/A	N/A	N/A	N/A
	3	Trivial				4	Trivial				**3**	**Trivial**								
Cognitive function ^‡^	919	-3.78 [4.16, -11.71]	41.8	P=0.0003	88	1007	-2.02 [5.32, -9.36]	46.43	P<0.0001	87	899	-4.14 [2.29, -10.57]	19.97	P=0.07	62	N/A	N/A	N/A	N/A	N/A
	3	Small				4	Small				3	Small								
Social function ^‡^	919	-11.31 [4.43, -27.05]	183.61	P<0.00001	95	1007	-4.38 [8.20, -16.97]	149.24	P<0.00001	93	911	-6.56 [2.03, -15.14]	41.72	P=0.02	75	N/A	N/A	N/A	N/A	N/A
	3	Medium				4	Trivial				3	Trivial								
Global QoL ^‡^	**1753**	**-7.26 [-5.88, -8.64]**	**3.85**	**P=0.15**	**48**	**1740**	**-4.94 [-2.95, -6.94]**	**1.04**	**P=0.25**	**28**	1758	-0.52 [4.67, -5.72]	21.2	P=0.0002	85	1489	**0.11 [1.58, -1.36]**	**1.37**	**P=0.24**	**27**
	**3**	**Small**				**3**	**Trivial**				4	Trivial				2	**Trivial**			
Dyspnea ^‡^	1753	-12.06 [-15.97, -8.15]	7.67	P=0.02	74	1740	-10.48 [-15.13, -5.84]	12.28	P=0.003	83	1692	-8.98 [-13.20, -4.76]	9.56	P=0.01	77	1489	-6.37 [-7.23, -5.52]	2.87	P=0.09	65
	3	Medium				3	Small				3	Small				2	Small			
Pain ^‡^	1753	-13.85 [-17.30, -10.40]	5.81	P=0.05	67	1845	-11.36 [-14.48, -8.23]	4.82	P=0.09	53	1853	-5.04 [-10.96, 0.87]	33.42	P<0.00001	86	1489	-5.76 [-12.71, 1.18]	23.29	P=0.0003	92
	3	Medium				4	Small				5	Small				2	Small			
Fatigue ^‡^	1753	-20.60 [-24.12, -17.08]	5.74	P=0.06	64	1841	-13.68 [-19.58, -7.78]	26.49	P=0.0001	86	1787	-9.27 [-15.27, -3.27]	27.38	P=0.0001	85	1489	-6.48 [-10.74, -2.21]	7.79	P=0.02	80
	3	Large				4	Medium				4	Small				2	Small			
Insomnia ^‡^	**919**	**-7.64 [-11.52, -3.76]**	**2.53**	**P=0.28**	**21**	**906**	**-4.48 [-8.34, -0.63]**		**P=0.22**	**35**	**852**	**-5.82 [-10.08, -1.56]**	**0.05**	**P=0.82**	**0**	N/A	N/A	N/A	N/A	N/A
	**3**	**Small**				**3**	**Small**				**2**	**Small**								
Anorexia ^‡^	919	-18.36 [-32.27, -4.45]	129.55	P=0.0005	87	1007	-6.71 [-16.85, 3.43	87.85	P=0.0002	85	**899**	**-4.29 [-9.73, 1.15]**	**8.92**	**P=0.21**	**36**	N/A	N/A	N/A	N/A	N/A
	3	Medium				4	Small				**3**	**Small**								
Nausea and Vomiting ^‡^	919	-10.43 [-19.51, -1.35]	56.71	P<0.0001	91	1007	-6.06 [-13.10, 0.97]	41.88	P<0.0001	86	899	-5.20 [-12.20, 1.81]	28.32	P=0.02	75	N/A	N/A	N/A	N/A	N/A
	3	Small				4	Small				3	Trivial								
Constipation ^‡^	**919**	**-4.27 [-7.21, -1.33]**	**1.48**	**P=0.48**	**0**	**1243**	**-5.42 [-8.26, -2.58]**	**3.22**	**P=0.20**	**38**	**804**	**-3.10 [-6.31, 0.10]**		**P=0.90**	**0**	N/A	N/A	N/A	N/A	N/A
	**3**	**Trivial**				**3**	**Small**				**2**	**Trivial**								
Diarrhea ^‡^	919	-12.42 [-20.99, -3.85]	43.7	P=0.01	77	906	-13.44 [-23.53, -3.34]	65.62	P=0.002	84	**804**	**-12.69 [-15.97, -9.41]**		**P=0.40**	**0**	N/A	N/A	N/A	N/A	N/A
	3	Small				3	Small				**2**	**Small**								
Financial ^‡^	**169**	**-7.18 [-13.71, -0.65]**	**1.06**	**P=0.30**	**6**	**167**	**-6.51 [-13.31, 0.29]**		**P=0.24**	**29**	N/A	N/A	N/A	N/A	N/A	N/A	N/A	N/A	N/A	N/A
	**2**	**Small**				**2**	**Small**													
Dysphagia ^§^	919	6.70 [-17.38, 30.77]	440.55	P<0.00001	98	1007	7.11 [-12.89, 27.10]	398.73	P<0.00001	97	899	-4.51 [-14.17, 5.14]	57.58	P=0.005	81	N/A	N/A	N/A	N/A	N/A
	3	Small				4	Small				3	Trivial								
Eating Problem ^§^	919	-5.85 [-34.21, 22.50]	616.02	P<0.00001	98	1007	-0.94 [-21.86, 19.97]	440.12	P<0.00001	97	899	-3.51 [-12.05, 5.04]	43.43	P=0.01	78	N/A	N/A	N/A	N/A	N/A
	3	Small				4	Trivial				3	Trivial								
Reflux ^§^	804	-14.01 [-39.88, 11.86]	339.53	P<0.00001	97	915	-5.21 [-19.44, 9.01]	148.13	P<0.00001	95	**788**	**-3.25 [-6.87, 0.37]**		**P=0.72**	**0**	N/A	N/A	N/A	N/A	N/A
	2	Medium				3	Trivial				**2**	**Trivial**								
Pain-OES18 ^§^	804	0.46 [-3.91, 4.84]	6.49	P=0.09	64	896	2.13 [-2.65, 6.92]	11.24	P=0.05	66	**788**	**0.31 [-3.08, 3.69]**	**1.14**	**P=0.29**	**9**	N/A	N/A	N/A	N/A	N/A
	2	Trivial				3	Trivial				**2**	**Trivial**								
Swallowing problem ^§^	919	-6.36 [-14.87, 2.15]	45.68	P=0.003	83	906	-5.05 [-13.39, 3.29]	43.04	0.005	81	804	-6.40 [-17.92, 5.12]	61.41	P=0.004	88	N/A	N/A	N/A	N/A	N/A
	3	Small				3	Small				2	Trivial								
Dry mouth ^§^	919	-5.82 [-11.64, -0.01]	13.73	P=0.13	51	906	-5.66 [-12.38, 1.06]	22.69	P=0.06	65	**804**	**-9.19 [-13.24, -5.13]**		**P=0.30**	**5**	N/A	N/A	N/A	N/A	N/A
	3	Small				3	Small				**2**	**Small**								
Taste problem ^§^	919	-10.26 [-26.13, 5.61]	181.5	P<0.00001	93	906	-6.75 [-16.15, 2.65]	54.91	P=0.006	81	**804**	**-8.42 [-12.46, -4.38]**		**P=0.28**	**14**	N/A	N/A	N/A	N/A	N/A
	3	Medium				3	Small				**2**	**Small**								
Cough problem ^§^	919	-15.35 [-25.20, -5.50]	56.49	P=0.02	76	906	-12.05 [-23.21, -0.88]	78.64	P=0.004	82	804	-11.01 [-18.89, -3.13]	19.56	P=0.13	55	N/A	N/A	N/A	N/A	N/A
	3	Medium				3	Medium				2	Medium								
Speech problem ^§^	**919**	**-9.94 [-12.68, -7.20]**		**P=0.15**	**48**	**906**	**-8.45 [-11.07, -5.84]**	**1.9**	**P=0.39**	**0**	870	-3.16 [-9.25, 2.92]	17.59	P=0.08	60	N/A	N/A	N/A	N/A	N/A
	**3**	**Small**				**3**	**Small**				3	Trivial								

*N/A* not applicable (this means that no two patient groups were available to derive a summary estimate of HRQL change by meta-analysis)

*MD* Mean difference / mean change

^†^The amount of patients in each analysis are the number of patients at baseline *and* the given follow-up time (e.g., the number of patients at 3 months follow-up represent the number of patients at baseline and the number of patients at 3 months follow-up, combined).

^‡^Mean difference; measured by the QLQ-C30

^§^Mean difference; measured by the QLQ-OES18

'Sufficiently' homogenous estimates are presented in bold (I^2^ ≤50% AND P≥ 0.1)

### Primary outcomes

The direction and clinical relevance of HRQL change at 3-month follow-up are shown in Table 5. Patients’ physical function, role function, and global QOL deteriorated. All symptoms in QLQ-C30 and dry mouth, cough problem, and speech problem in QLQ-OES18 increased. However, the direction for other outcomes (three functional scales in QLQ-C30, four symptom scales, and two single items in QLQ-OES18) at 3-month follow-up were too heterogeneous to interpret.

**Table 5:** Main analysis for the direction, clinical relevance, and duration of change in 24 HRQL outcomes after MIE

HRQL outcome	HRQL change at 3-month follow-up				HRQL change at 3-, 6–12-,and/or 24 month follow-ups	
	Number of patients^†^ (Groups)	Amount of heterogeneity^‡^	Direction of HRQL change^§,¶^	Clinical relevance of HRQL change^\\,††^	Direction of HRQL change^§,¶,‡‡^	Duration of HRQL change^§§^
Physical function	1753 (3)	Substantial	Deterioration	Unclear	Deterioration	Unclear
Role function	919 (3)	Considerable	Deterioration	Unclear	Unclear	Unclear
Emotional function	919 (3)	Considerable	Unclear	Unclear	No change	No change
Cognitive function	919 (3)	Considerable	Unclear	Unclear	Unclear	Unclear
Social function	919 (3)	Considerable	Unclear	Unclear	Unclear	Unclear
Global QoL	1753 (3)	Moderate	Deterioration	Small	Deterioration	6 months
Dyspnea	1753 (3)	Substantial	Increase	Unclear	Increase	Unclear
Pain	1753 (3)	Substantial	Increase	Unclear	Increase	Unclear
Fatigue	1753 (3)	Substantial	Increase	Unclear	Increase	Unclear
Insomnia	919 (3)	Low	Increase	Small	Increase	>12 months^¶¶^
Anorexia	919 (3)	Considerable	Increase	Unclear	Unclear	Unclear
Nausea and Vomiting	919 (3)	Considerable	Increase	Unclear	Unclear	Unclear
Constipation	919 (3)	Low	Increase	Trivial	Increase	12 months
Diarrhea	919 (3)	Considerable	Increase	Unclear	Increase	Unclear
Financial	169 (2)	Low	Increase	Small	N/A	N/A
Dysphagia	919 (3)	Considerable	Unclear	Unclear	Unclear	Unclear
Eating Problem	919 (3)	Considerable	Unclear	Unclear	Unclear	Unclear
Reflux	804 (2)	Considerable	Unclear	Unclear	Unclear	Unclear
Pain-OES18	804 (2)	Substantial	Unclear	Unclear	Unclear	Unclear
Swallowing problem	919 (3)	Considerable	Unclear	Unclear	Unclear	Unclear
Dry mouth	919 (3)	Substantial	Increase	Unclear	Unclear	Unclear
Taste problem	919 (3)	Considerable	Unclear	Unclear	Unclear	Unclear
Cough problem	919 (3)	Considerable	Increase	Unclear	Increase	Unclear
Speech problem	919 (3)	Moderate	Increase	Small	Increase	12 months

^†^The number of patients included at baseline and the number of patients included at 3-month follow-up, combined

^‡^We used the *I*^2^ statistic to describe the percentage of inconsistency attributable to heterogeneity and not chance. An *I*^2^ of <30% represents low heterogeneity, 30–50% moderate, 50–75% substantial, and 75–100% considerable heterogeneity

^§^The direction of HRQL change was clear if the estimate was “sufficiently” homogenous (i.e., *χ*^2^ *P* ≥ 0.1 and *I*^2^ low or moderate) or, when the estimate was not “sufficiently” homogenous, if both the summary estimate and confidence intervals reported the same direction [e.g., − 5,00 (− 10,00; − 2,00)]

For functioning scores, “deterioration” indicates that the follow-up scores were lower than baseline scores. For symptom scales, “increase” indicates that the follow-up scores were higher than baseline scores

^\\^The clinical relevance of HRQL change was clear if the estimate was “sufficiently” homogenous (i.e., *χ*^2^ *P* ≥ 0.1 and *I*^2^ low or moderate)

^††^A large change indicates a clear clinical relevance. A medium change indicates a clinical relevance, but to a lesser extent. A small change indicates a subtle but nevertheless clinically relevant effect. A trivial change indicates either a change of unlikely clinical relevance, or no change

^‡‡^The direction of HRQL change was clear if at least three estimates of change were obtained, two of which were “sufficiently” homogenous (i.e., *χ*^2^ *P* ≥ 0.1 and *I*^2^ low or moderate), and if the summary estimates showed the same direction of change. If none of the estimates were “sufficiently” homogenous, we determined that the direction of HRQL change was clear if the summary estimates and confidence intervals at 3-, 6-, 9-, and 12-month follow-ups showed the same direction of change [e.g., − 5,00 (− 10,00; − 2,00)]

^§§^The duration of HRQL change was clear if at least three estimates of HRQL change were obtained (e.g., at 3-, 9-, and 12-month follow-ups). Two of these estimates had to be “sufficiently” homogenous (i.e., *χ*^2^ *P* ≥ 0.1 and *I*^2^ low or moderate)

The duration of HRQL change lasted longer if the clinical relevance of the last sufficiently homogenous estimate was not trivial or if subsequent estimates were not “sufficiently homogenous”

The global QOL, insomnia, financial problem, and speech problem changes were small, which showed clinical relevance at 3-month follow-up. However, other HRQL outcomes (five functional scales, three general symptom scales and four single items in QLQ-C30, and four symptom scales and four single items in QLQ-OES 18) did not show clinical relevance.

### Secondary outcomes

The direction and duration of HRQL change at 3-, 6-, 12-, and/or 24-month follow-ups are reported in Table 5. The directions of patients’ physical function and global QOL deteriorated, and emotional function had no change. The directions of dyspnea, pain, fatigue, insomnia, constipation, diarrhea, cough problem, and speech problem were increased. However, the direction for other outcomes (three functional scales and two single items in QLQ-C30 and four symptom scales and three single items in QLQ-OES 18) at 3-, 6-, 12-, and/or 24-month follow-ups were too heterogeneous to interpret.

The deterioration in global function lasted 6 months after surgery. The increase in constipation and speech problem lasted 12 months after surgery. And, symptoms of insomnia increased more than 12 months after surgery. However, the duration for other outcomes (four functional scales, three general symptom scales and three single items in QLQ-C30, and four symptom scales and four single items in QLQ-OES 18) were too heterogeneous to interpret.

## Discussion

Several studies had shown that open esophagectomy has a negative impact on almost all aspects of HRQL, and it would take 9–12 months to return to levels before operation [29–31]. Others reported that HRQL recovered more quickly after MIE than OE [15, 17–20].

This is the first meta-analysis to estimate the clinical relevance and duration of change for 24 HRQL outcomes from short- to long-term after MIE. After MIE, patients’ emotional function has no change from short- to mid-term follow-up; global QOL deteriorated only at short-term follow-up. Symptoms of constipation and speech problem increased from short- to mid-term follow-up, and insomnia increased up to long-term follow-up. In addition, global QOL, most functional scales, and most symptoms have negative change at short-term follow-up and keep the trend to mid- and/or long-term follow-up. However, the clinical relevance and the duration of most change cannot be interpreted because of huge heterogeneity.

The clinical heterogeneity of the studies focusing on HRQL after esophagectomy has been presented in many studies. Levenstein et al. [32] assessed HRQL by an international comparison. The study showed that HRQL varied from one country to another because of differences in social, cultural, medical systems, race, family structure, and/or economic determinants with relevance to the patient-physician relationship, patient education, and therapeutic decision making, and other factors. Comorbidity, tumors located in the middle or upper esophagus, SCC histology, tumors in stages III and IV have been reported to be associated with worse HRQL. And, patients with early disease stages had better HRQL than those with more locally advanced disease [33, 34].

It is still controversial whether patients receiving neoadjuvant chemotherapy or chemoradiotherapy had worse HRQL than those who underwent esophagectomy alone. Blazeby et al. [35] reported that patients who received palliative treatment had significantly worse pain, fatigue, appetite loss, constipation, and dysphagia. Other studies had shown that preoperative chemotherapy or chemoradiotherapy had a temporarily negative impact on HRQL, which returned to baseline levels before surgery, and recovery of HRQL after esophagectomy was not impaired by neoadjuvant treatment [29, 36]. In contrast, Ariga et al. [37] observed that patients with squamous cell carcinoma who underwent definitive chemoradiation had similar general HRQL scores and lower diarrhea, appetite loss, and eating problem scores than those who had undergone surgery alone.

The surgical approach (MIE vs. OE) and the length of the postoperative time period have been presented that had a positive impact on patients’ postoperative QOL (global QOL, physical function, fatigue symptoms, pain symptoms, and dyspnea symptoms) [19]. And conservative, non-definitive treatments such as endoscopic treatment may cause more fear of recurrence than esophagectomy, which may have negative effect on HRQL [38].

It is impossible, up to now, to get enough homogeneous studies to analyze HRQL after MIE [13, 14]. In other words, the heterogeneities of studies included in our meta-analysis were unavoidable. Studies from five different countries were included. The tumor histology and tumor stage were different from one study to another. Whether neoadjuvant treatments were performed varied among the included studies.

Our study has some limitations. First, we selected more retrospective studies, and only one randomized controlled trial. Only one study aimed to assess the impact of MIE on HRQL, others aimed to compare HRQL between MIE an OE. Second, there were not enough studies available to investigate the influence of MIE on HRQL [39]. Third, we chose studies reported in English, but omitted non-English studies [40]. All these might lead to bias. Therefore, more randomized controlled trials are needed to validate HRQL after MIE.

## Conclusions

This meta-analysis shows that the emotional function had no change after operation. The global function became worse during the early postoperative period; the symptoms of constipation, speech problem, and insomnia increased for a long time after operation.

## Abbreviations

EORTC: European Organization for Research and Treatment of Cancer
HRQOL: Health-related quality of life
MIE: Minimally invasive esophagectomy
OE: Open esophagectomy
QLQ: Quality of life questionnaire
